# A Game-Theory-Based Approach to Promoting Health Policy among Minorities

**DOI:** 10.3390/ijerph20054335

**Published:** 2023-02-28

**Authors:** Chen Cohen, Lilach Rinot Levavi

**Affiliations:** Department of Public Policy and Management, Ben-Gurion University of the Negev, Beer-Sheva 84105, Israel

**Keywords:** COVID-19 vaccination, game theory, public trust, conservative minorities, Bedouin, health literacy

## Abstract

The importance of designing policy measures that government and other public bodies apply to different populations has been escalating in recent decades. This study seeks the best way to induce conservative minority groups to cooperate with healthcare policy. The case study focuses on the Bedouin population of Israel and its willingness to accept COVID-19 vaccination. The study is based on vaccination data from the Israel Ministry of Health for the country’s entire Bedouin population, twenty-four semi-structured in-depth interviews with relevant key stakeholders, and the use of game-theory tools to profile the players, the utility functions, and various equilibrium combinations. By comparing the groups and integrating game-theory tools into the process, we reveal variables that may affect healthcare processes among conservative minority communities. Finally, cross-tabulating the results with the interview findings strengthens the insights and allows a culturally adjusted policy to be adopted. The different starting points of different minority populations have implications for the design of requisite policies in both the short and the long terms. The analysis of the game allowed us to indicate the strategy that policymakers should adopt in consideration of variables that should be taken into account in order to improve cooperation and the ability to apply policy. To increase vaccination rates among conservative minority communities in general and the Bedouin population in particular, trust in the government must be increased in the long term. In the short term, trust in the medical profession must be increased, and also health literacy.

## 1. Introduction

### 1.1. Background

The coronavirus outbreak has demonstrated the importance of public compliance with government health instructions. The most effective way to obtain protection against serious infection, hospitalization, and death from COVID-19 is vaccination [1]. In a large study conducted in Israel, vaccine effectiveness against these consequences was found to exceed 96% in all age groups, including older adults [2]. Launching its COVID-19 vaccination campaign on December 20, 2020, the Government of Israel urged the population to be vaccinated [3]. The country rose to the challenge; by the end of that year, Israel, with a population of 9.3 million, had obtained more COVID-19 vaccine doses than all countries except China, the United States, and the United Kingdom [4]. Notwithstanding this impressive achievement, many citizens of the country’s Arab minority ignored the government’s calls to be vaccinated despite regular and repeated urgings on multiple media and by public figures including local government officials, [5,6] significant efforts to work with local healthcare and religious leaders, the deployment of mobile clinics, and extended hours of operation at vaccination sites [7]. Vaccine uptake was significantly low among one specific Arab ethnic group in Israeli society, the Bedouin of the south (the Negev). 

In this article, we seek an optimal way to encourage conservative minority communities to cooperate with a Western healthcare policy in regard to vaccination. The case study is the Bedouin population of Israel, differentiated by those in the south and those in the north. The method is based on three main research tools: vaccination data from the Israel Ministry of Health for the Bedouin population (southern and northern), 24 semi-structured in-depth interviews with key personalities in the Bedouin society (such as doctors, officials, and functionaries), and game-theory tools that help to profile possible equilibria and ways of converging toward them. By constructing a model on the basis of the variables that we found, we identified measures and processes that policymakers should carry out in the long and the short terms.

Our research question is addressed to two main aspects that deserve examination: the role of the unique characteristics of the Bedouin population in Israel in their willingness to receive COVID-19 vaccination, and factors that may whet this motivation, by comparing the southern Bedouin with those in the north. Two variables were used for the comparison: extent of trust in the government and professionals, and health literacy. We also wished to develop a model that would help identify the variables that influence convergence to possible equilibria in the field.

This is a mixed-methods study, using official vaccination data, interviews, and game-theory tools to identify optimally the variables of relevance that may shift the research population toward a more efficient equilibrium. The integration of game-theory tools will make it possible to improve healthcare processes among the Bedouin and among minority groups at large.

### 1.2. Bedouin Communities in Israel

Israel’s population is estimated at 9.291 million—21.1% Arabs, 73.9% Jews, and 5.0% others [8]. The Bedouin are a subgroup within the Arab minority of Israel, with cultural, historical, social, and political uniqueness.

Bedouin in Israel are traditionally a semi-nomadic population that comprises about 3% of Israel’s population [9]. During the past half-century, as a result of intensive exposure to Western culture, values, and norms, this population has experienced a rapid transition from traditional to modern life. Despite these changes, Bedouin society remains characterised by collectivist cultural orientations as defined by some researchers [10,11,12].

The focus of this article is on the Bedouin populations of the north and the south of Israel. Although both communities are characterized by low socioeconomic status, their characteristics differ in significant ways: 

The larger of the two Bedouin communities is that in the south (the Negev), at ~280,000 people [9]. Some 70% of this population lives in 19 recognized localities; the rest belong to 22 tribes that live outside recognized localities. Although this traditionally nomadic tribal community has transitioned into a primarily urban population within a single generation, some 40% of its members still live in rural areas under semi-nomadic conditions. The Bedouin community is characterized by (1) the change of its lifestyle from seminomadic and rural to semi-urban, (2) low educational attainment relative to the entire Israeli population, (3) crowded living conditions among extended families, (4) the need to cope with daily problems amid incomplete connection to environmental infrastructures such as water, electricity (about 40% of these citizens live without consistent access to electricity), and the internet [13], as well as poor access to public transportation, and (5) its relationship with the State of Israel, which is rather tense due to issues of discrimination and the legal right to own land [4,13,14,15]. 

**Figure 1:**
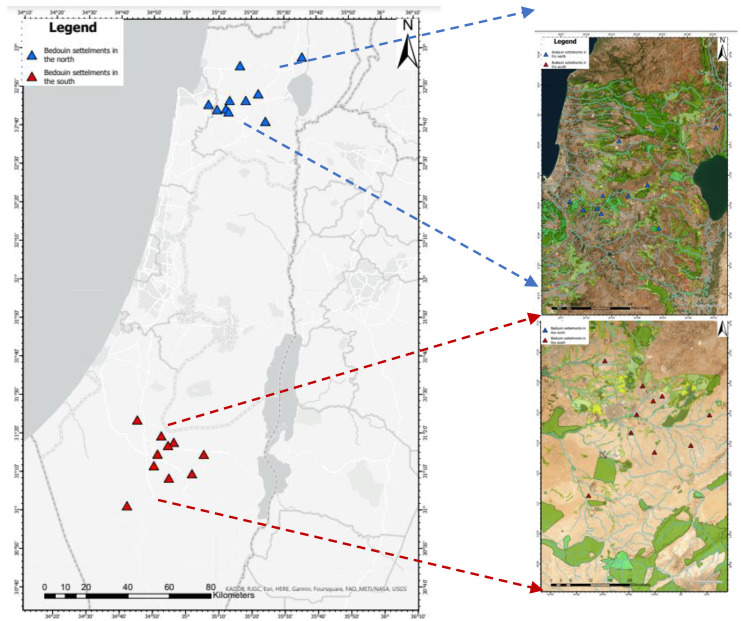
Geographical location and distribution of Bedouin settlements in the areas studied.

The northern Bedouin community is located in the Galilee and comprises ~70,000 people in 16 permanently recognized villages and a smaller number in unrecognized villages. This community is well along in transitioning from nomadic life to the local urban way of living. It is characterized by (1) close and cooperative relations with Jewish society in the Galilee, manifested in (2) a high percentage of youth who join the army (two-thirds of Bedouin soldiers come from the northern community, as against one-third from the southern population, and (3) advanced environmental infrastructure and good connections to water, electricity, and public transportation [16,17,18]. The geographic location and distribution of Bedouin settlements in the north and south of Israel, for the purpose of this study, are shown in Figure 1.

### 1.3. The Attitude of the Bedouin Community toward Vaccines 

Vaccination coverage in Israel is high [19] (Somekh et al., 2022). Israel ranks among the top half of Western countries in this respect. Routine vaccinations are given under the National Health Insurance Law [3]. The Ministry of Health updates the vaccination program regularly on the basis of recommendations from an advisory committee on vaccinations and infectious diseases. Preventive care, including routine vaccinations, is provided by a network of governmental well-baby clinics [20]. Very high coverage percentages are found among the Bedouin in the recommended vaccination regime against children’s diseases [20,21,22]. An intervention program funded by the Ministry of Health was associated with a marked increase in vaccination coverage in Bedouin children—from 53% to 90% at age 2—and a concomitant decrease in vaccine-preventable infectious diseases [23]. The situation regarding COVID-19 vaccination is significantly different. The vaccination rate in Arab localities, and particularly among the Bedouin, fell short of that of the general population [7,13,15]. In a survey conducted by the Red Cross, it was found that around 60% of southern Bedouin had no interest in being vaccinated [13,24]. 

These data underscore the need to investigate the attitudes of Bedouin society toward vaccination and the community’s response to instructions from the government and healthcare professionals. Although our concern in this article centers on the attitude toward COVID-19 vaccination, our goal is to generalize the findings into a full picture of the question of vaccination and cooperation with the state among conservative minority groups. 

An important predictor of compliance with vaccination guidelines among conservative minority groups in general, and the Bedouin community in particular, is the group’s level of trust in the government. 

### 1.4. Trust as a Promoter of Public Policy and Healthcare Professionals

Citizens’ willingness to follow instructions from the state depends on their trust in the state and its authorities. This attests in crisis situations, particularly health crises, more than it does in normal times [25,26]. A community’s level of trust can determine its intention to accept key public policies, affecting how well the country manages health crises such as pandemics [26,27]. Several studies have shown a link between trust and success in several actions related to the COVID-19 pandemic, such as vaccination, social distancing, and masking [26,28]. Minority groups often trust the government less than the majority for reasons including their lack of integration and representation in government institutions, and limited political efficacy [29]. In Israel, the mistrust of majority intentions in Arab society is intensified by the long-enduring Israel–Palestinian conflict [30].

As for healthcare professionals, [31] demonstrate the crucial role of doctor–patient communication in building patients’ trust in doctors, which in turn positively impacts vaccination attitudes and H1N1 vaccination behavior. Harrington et al. [32] show that doctors and healthcare providers are highly trusted. Kalagy et al. [33] found that members of the Arab minority in Israel listen first to doctors who are experts in infectious diseases and second to family doctors with regard to messages about the COVID-19 pandemic. The most influential sources for parents’ decisions to vaccinate their children are nurses at the well-baby clinics (which are primarily staffed by public-health nurses and engage in intensive outreach for vaccinations and general healthcare for children up to age 6) [34]. This was also evidenced in a nationwide polio vaccine campaign in 2013, with public-health nurses as key to the successful elimination of transmission [22]. 

Other studies, however, find lower levels of trust in healthcare systems and professionals among religious and ethnic minorities [35]. 

### 1.5. Health Literacy

The World Health Organization defines health literacy as “the cognitive and social skills which determine the motivation and ability of individuals to gain access to, understand and use information in ways which promote and maintain good health” [36] World health organization (N.D). Low health literacy is associated with older adults, adolescents, racial and ethnic minorities, limited language proficiency, limited education, and low income [37,38,39]. Its adverse health consequences are experienced by most minority groups [38,40] and are observable in areas as diverse as cancer research and clinical trials, mental health, oral health, and sexual risk behaviors [41,42]. 

Health literacy is lower in disempowered and less affluent communities than elsewhere; thus, people in these settings tend to misinterpret or fail to receive health authorities’ instructions and warnings [43]. Dewalt et al. [44], reviewing the relationship between literacy and health outcomes, found an association between low health literacy and worse health outcomes: less knowledge, more disease markers, higher morbidity indices, poorer general health status, and reduced utilization of healthcare resources.

The cruciality of health literacy was demonstrated in its role during the COVID-19 pandemic in enabling citizens to deal with health information regarding the illness [45,46,47]. Conversely, inadequate health literacy and digital health literacy were associated with consumption of false information, contributing directly to the dissemination of misleading content online in regard to COVID-19 [48,49].

Given that the Arab population in Israel exhibits poor health literacy [21,50,51], below we ask whether this deficiency may have contributed to the low COVID-19 vaccination rates that were found among the Bedouin population.

### 1.6. The Game-Theory Approach 

Game theory uses mathematical models to predict the behavior of rational players in situations of conflict or cooperation and analyzes how their inter-effects channel their decisions and beget the final outcome. Games may be analyzed under different states of information (complete and incomplete), degrees of symmetry (symmetric and asymmetric games), and types of interaction (simultaneous and serial).

Game-theory models have been used to solve environmental dilemmas pertaining to the incentivization and promotion of green construction [52,53], sustainable municipal solid-waste disposal [54], and so on. Cohen et al. [55] show that mistrust and lack of cooperation steer such a game to a passive equilibrium in which no potential player in a recycling process agrees to participate.

Scholars use game-theory models to improve healthcare quality, management, performance, and productivity [56,57,58], and to encourage vaccination behavior [59,60]. Thus, we present an analysis of simultaneous interaction among asymmetric players who differ in their utility functions and play under conditions of complete information. 

Next, we map the relevant players and the parameters that affect them in search of an effective mechanism for the implementation of an appropriate policy that will promote vaccination in Israel’s Bedouin society. Our innovation is reflected in our processing of data from the qualitative section into input in modeling carried out with the help of game theory. Novel as well are the findings of our analysis of the model, which help to identify variables and measures for this social-health process in particular and among minority groups in general.

## 2. Materials and Methods

### 2.1. Methods and Procedure

In this study, we combined a mixed quantitative and qualitative method with the mathematical model of a game situation. Our assumption was that the qualitative research would provide a basis for the quantitative work in a way that would reinforce the validity of the findings and the model developed [61]. 

**Figure 2:**
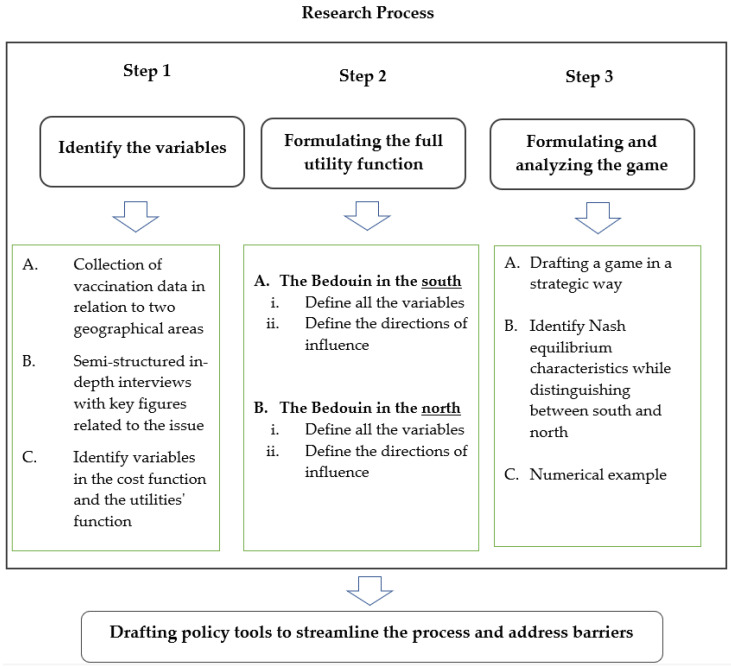
The research process.

The study took place in three steps (Figure 2) that combine three main research tools:

1. Gathering and analyzing statistical data about vaccination among Bedouin citizens in northern and southern Israel;
2. Semi-structured in-depth interviews with 24 key players in regard to the research topic;
3. Constructing and analyzing a model derived from game theory.

The diagram below illustrates the third research method and the overall research process:

Step 1: We identified and estimated the variables and parameters for all players. To determine the vaccination percentages among Bedouin in the north and south and compare them with those of the general population, we analyzed data from the Ministry of Health database. We gathered the data for the Bedouin population by locating all Bedouin settlements in the north and south and consolidating their data into one central figure. To assess the attitudes of the Bedouin toward routine vaccinations and COVID-19 vaccination, we complemented this information by conducting 24 semi-structured in-depth interviews to attain a precise definition of the variables in the cost and utility functions.

Step 2: We formulated full utility functions for all players, profiling the players’ parameters and utilities on the basis of the literature sources and the interviews. The interviews yielded specific variables for these profiles.

After identifying the variables, we defined for each player the ones that affected his or her utility function, their intensity, and the type of relation (positive or negative) in question. The quantitative research tools included those of game theory.

Step 3: We presented a simultaneous strategy game with full information. First, the equilibria in pure strategies between citizens and the government were presented. Second, we considered the possibility of a mixed-strategy equilibrium and showed that moving from one combination of strategies (in pure equilibrium) to another would be contingent upon being in a mixed-strategy equilibrium. The various equilibria were analyzed for settlements in both the northern and the southern regions. Finally, a numerical example illustrating all the findings was presented.

In this study, we attempted to determine the optimal platform for the mobilization of conservative minority communities behind Western healthcare policies relating to vaccination. The case study chosen was the Bedouin society of Israel, with a distinction made between that in southern Israel and that in the north.

Below we present the findings in the order of the three main research tools that we used to gather the data. First, we show vaccination percentages determined by the Ministry of Health website after the requisite statistical testing. Then, we report the main themes that emerged from the interviews conducted and the variables mapped. Finally, we represent a game in the form of a strategy that will allow us to identify combinations at equilibrium. The overarching aim is to identify the variables that will make convergence to a more efficient equilibrium possible.

### 2.2. Gathering the Ministry of Health Data

In January 2022, we harvested data from the Ministry of Health website on rates of take-up of all three doses of COVID-19 vaccination among the inhabitants of 20 Bedouin localities, divided geographically into northern and southern Israel (10 localities in each). These settlements are home to 283,135 residents; the others belong to the general population of the country.

**Figure 3:**
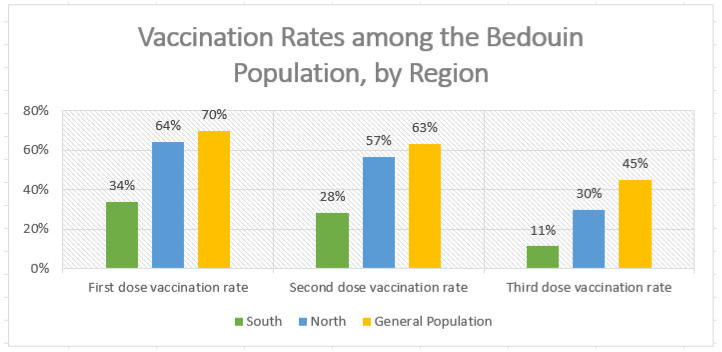
Vaccination rates among the Bedouin population, by region.

Figure 3 and Table 1 compares the Bedouin vaccination rates with those of the general population for each of the three doses of vaccine that were administered in Israel at the time the data were gathered.

The first-dose vaccination rate of division N (M = 0.63, SD = 0.06, *n* = 10) is hypothesized as greater than the first-dose vaccination rate of division S (M = 0.26, SD = 0.15, *n* = 10).

The difference is significant: t (18) = −7.32, *p* < 0.001.

The second dose vaccination rate of division N (M = 0.56, SD = 0.05, *n* = 10) is hypothesized as greater than the first dose vaccination rate of division S (M = 0.22, SD = 0.13, *n* = 10).

The difference is significant: t (18) = −7.76, *p* < 0.001.

The third dose vaccination rate of division **N** (M = 0.29, SD = 0.05, *n* = 10) is hypothesized as greater than the first dose vaccination rate of division **S** (M = 0.08, SD = 0.07, *n* = 10).

The difference is significant: t (18) = −7.98, *p* < 0.001.

### 2.3. Findings of Interviews 

To investigate the low vaccination rates that we found among the southern Bedouin population in Step 1, relative to the general population and particularly to the northern Bedouin population, we examined the inhibitions that lead to this outcome.

Semi-structured interviews with key figures gave us a closer look at the topic investigated by allowing local voices to be heard. The interview was composed of a systematic set of questions that could be adjusted based on the interviewer–interviewee dynamic that took shape [61,62] Twenty-four interviews were conducted and transcribed in January–March 2022 with various professionals and public figures: these included 8 northern Bedouin (4 physicians and nurses working for healthcare systems, 4 social activists and public figures), and 8 southern Bedouin (2 physicians, 5 social activists and public figures, 1 Muslim religious leader). Interviewed as well were 3 public-health executives in the healthcare system. Given the diversity of the Arab population, we conducted 4 additional interviews—with 2 public figures from the Druze community, 1 from the Circassian community, and 1 Christian Arab physician—in order to gain further insights from their views on the issue. Altogether, 7 women and 16 men were interviewed, most anonymously at their request. 

The questions they were asked revolved around five main aspects:

1. What is the rate of response to COVID-19 vaccination among the Bedouin population?
2. What parameters, in your opinion, affect the vaccination rate in Bedouin society? (Culture and literacy were noted here.)
3. How did Bedouin society receive the government’s instructions on vaccination?
4. How, in your opinion, did the state deploy for the current health crisis, with emphasis on communication with the Bedouin population?
5. Which personalities in the community may have been helpful or unhelpful in accepting the government’s instructions during the crisis?

The responses yielded several recurrent themes:

**Low COVID-19 vaccination rates.** Despite strong awareness and responsiveness to routine vaccinations, the COVID-19 vaccines met with significant resistance, reinforced by the intensive spread of fake news in Arabic-language social media. 

“We tried all kinds of campaigns in the Bedouin sector. […] One of the problems we had was that women were afraid to be vaccinated due to fertility issues; this created round after round of problems” (Interview no. 1, with a healthcare official in the Southern District).

There are lots of initiatives. “[…] They wanted people to be vaccinated, encouraged them to do so, [told them] not to be afraid of the vaccines, it is conducted in the schools, too, and in the education system we worked hard to make parents aware that the vaccine does not hurt children. […] Lots was implemented to enhance awareness, but it did not help” (Interview no. 2, school principal).

**Poor health literacy.** In his interview, a senior healthcare official in the south raised an important issue relating to knowledge. This aspect includes two perspectives, he said. From the citizens’ side, poor health literacy attests in gaps in medical knowledge due to ignorance and digitization. From the side of the healthcare system, there is a cultural mismatch with the tribal Bedouin society, with emphasis on language.

“It is a matter of culture. […] There are lots of far-flung settlements, lots of cultural and language difficulties. Most of our material is in Hebrew and English; making it accessible in Arabic is no simple matter. You have to make sure the whole campaign is accompanied by advertising in the right language” (Interview no. 1, with a healthcare official in the Southern District). 

**Distrust in the government.** The distrust issue recurred throughout the interviews. It originates in preconceptions and suspicion of the governmental system and the sincerity of its intentions in vaccinating the population: 

“Religion is less central in Bedouin society; rather, it is tradition, preconceptions: […] [The authorities] want to impair our fertility because [they consider us] a demographic threat” (Interview no. 3, public figure, attorney).

The interviews reflected significant distrust of the state, including the Ministry of Health and its representatives. Trust in healthcare workers, however, is strong, meaning specialist doctors, family doctors, nurses, and staff of the well-baby clinics where vaccinations are administered in ordinary times.

**Cultural gaps.** The interviews also revealed cultural gaps rooted in Bedouin clan values, attested at three meaningful levels:

1. Selection of healthcare staff:

“Clan struggles have something to do with it. If the doctor comes from the rival clan, people will not follow him. It is a difficult mentality. Family rivalries trump considerations of public welfare. As for the vaccines, it is clear that if one family goes out to be vaccinated its rival will do the opposite on purpose” (Interview no. 2, school principal).

2. Figures of authority who can convey medical messages to the Bedouin community. When interviewees were asked to identify these figures, they emphasized doctors:

“It is different from Haredi communities, which do what the rabbi says. In Bedouin society, you have the clan and each clan and each family has its influencer. Therefore, it is a different social structure. […] If only there were one clear central figure” (Interview no. 2, school principal).

“The ones who can influence are the doctors. No sheikh or family head will persuade me about something like this—it is not his field, no one will take him seriously” (Interview no. 4, Bedouin doctor).

3. Chaotic guidelines. The interviewees mentioned the authorities’ confusing messages and often-changing instructions as a significant factor in the failure to present a clear plan. In addition, the simple and placatory messages that appear in social media make it difficult for official messages to gain traction. 

“The fear is connected with uncertainty, confusion, and media reportage that encourages even more fear and distrust also raise the barriers. […] Lots of people hesitate to become vaccinated, do not become vaccinated, and do not mask when they have guests” (Interview no. 5, Bedouin midwife).

**Leadership crisis.** The leadership crisis in Israel’s Arab society generally, and that in Bedouin society particularly, abetted the COVID-19 crisis, as a member of Bedouin society in the south explains:

“There is no leadership in Arab society, no classical leadership. The council chairman, whom no one likes? Clerics? The village mayor? You will not find important doctors and teachers—they influence their families but not at the level of the entire community. There is no leadership in the Israeli Arab sector. There is no Bedouin leadership” (Interview no. 6, Bedouin male nurse).

### 2.4. Game Theory

By using the first two research tools, we were able to define and formulate parameters that would elicit definitions of the players’ utility functions. Strategically, the game was set up for two players: Bedouin citizens and the government. We also defined each player’s action strategies and utility functions for each combination of strategies. Due to differences in characteristics between the northern and southern Bedouin populations, we highlighted the difference by using partial derivatives. We identified equilibria in pure and mixed strategies after fully characterizing the game and illustrate them below in a numerical example that shows the differences between the regions. Finally, we weighed policy measures that might change the equilibrium in the game toward greater efficacy, thus increasing the vaccination rate among minorities, viz., the Bedouin.

Defining the Model

The players in the model are the following:

(a) citizens;
(b) government.

The citizens’ strategies are the following:

(a) Receive a COVID-19 vaccine.
(b) Do not receive a COVID-19 vaccine.

The government’s strategies are the following:

Make all necessary efforts to induce Bedouin citizens to receive a vaccine (marked as “To invest”) or choose not to invest in the process (“Not to invest”).

(a) To invest;
(b) Not to invest

As a first step, we describe the mechanics-adapted functions of each player and then explain their components and meaning, with which we represent the utility functions. All parameters included are defined in Table 2. 

#### 2.4.1. The Citizens’ Utility Function Comprises Their Utility from Vaccination Less Their Vac-Cination Costs, as Follows

(1) $$\;U_{C}=V_{C}\;(T_{HP},T_{G}{}_{\;})-C_{C}\left(H_{L}\right)$$

$\left(A\right)\frac{\partial V_{C}\;\left(T_{HP},T_{G}\right)}{\partial T_{HP}}>0\;;\;\left(B\right)\frac{\partial V_{C}\;\left(T_{HP},T_{G}\right)}{\partial T_{G}}>0;\;\left(C\right)\;\frac{\partial C_{C}\left(H_{L}\right)}{\partial H_{L}\;}<0.\;$

Citizens’ subjective utility from being vaccinated is positively contingent upon the level of trust (of both types *A* and *B*). That is, an increase in citizens’ trust in healthcare professionals increases the utility they gain by being vaccinated, as does an increase in their trust in the government.

The citizens’ cost function is adversely affected by health literacy. Thus, the stronger their health literacy is, the less effort it will take to persuade them to be vaccinated (*C*).

#### 2.4.2. The Government’s Utility Function Consists of Its Utility from Vaccinating Less Its Vac-Cination Costs, as Follows

(2) $$\;\;\;\;U_{G}=V_{G}\;(n_{v},T_{G})-C_{G}\left(H_{L}\right)$$

$\left(A\right)\frac{\partial V_{G}(n_{v},\;T_{G})}{\partial n_{v}}>0\;;\;\left(B\right)\frac{\partial V_{G}(n_{v},T_{G})}{\partial T_{G}}>0;\;\left(C\right)\frac{\partial C_{G}\left(H_{L}\right)}{\partial H_{L}}<0.\;$

The government’s utility from vaccinating citizens is positively contingent upon the number of citizens vaccinated (*A*) and the level of their trust in the government (*B*). The more citizens are vaccinated, the lower the morbidity rate will be, the better the government’s reputation, and the better its public image in all variables and in maintaining routine and economic life. All of these bolster the positive relation between the number of citizens vaccinated and the positive utility that the government gains by its citizens’ being vaccinated. 

The very act of vaccination is construed as an expression of citizens’ trust in the government. The more trust there is, the better the government’s image and the connection between the two. Processes that entail cooperation also become more efficient. Conversely, the cost to the government is adversely affected by health literacy, meaning that the higher the public’s level of health literacy is, the less cost the government needs to incur (*C*).

## 3. The Game

We present the players’ Nash equilibrium strategy in a simultaneous game with complete information as shown in Table 3.

We now test for the existence of an equilibrium in each of the four possible combinations (A, B, C, D) in pure strategies:

(3) $$R:\;U_{C}^{A}>U_{C}^{B}>0>U_{C}^{C}$$

Explanation of the utilities: Citizens maximize their utility when the government chooses to invest in the process and when citizens choose to be vaccinated and to cooperate ($U_{C}^{A}$). The second-greatest utility is obtained when the government invests effort in cooperation but citizens do not receive vaccinations (perhaps due to distrust in the government as the source of information) ($U_{C}^{B}$). Insofar as the government does not invest effort in cooperation and citizens do not receive vaccination, the citizens’ utility is normalized to zero. (This is the benchmark.) The worst situation for citizens is when the government does not invest in cooperation but they do receive vaccination. The utility to citizens is minimal because it is assumed that if citizens receive vaccination but not due to cooperation with and trust in the state institutions, and without understanding the importance of being vaccinated, the situation and the very act of vaccination will come at a steep cost ($U_{C}^{C}$).

(4) $$M:\;U_{G}^{A}>U_{G}^{C}>0>U_{G}^{B}$$

Explanation of the utilities: The government maximizes its utility when it invests in the process and when citizens choose to receive vaccination ($U_{G}^{A}$). Its second-greatest utility is attained when it invests efforts but citizens do not receive vaccination. In this situation, the government is spared the cost of cooperation but gains in three ways: good reputation, positive public image, and continuation of routine life ($U_{G}^{C}$). This situation ranks only second in prioritizing the government’s costs because it is assumed that vaccination based on cooperation and trust strengthens the government’s relations with its citizens, helping to reduce future costs. Vaccination without cooperation, in contrast, may weaken the relationship and impair the government’s future utility. Insofar as the government does not invest effort and citizens do not receive vaccination, the government’s utility is normalized to zero (the benchmark). The government is worst off when it invests effort but citizens choose not to be vaccinated. In this situation, the government absorbs all the costs of its effort to induce cooperation but gains no utility from citizens’ vaccination ($U_{G}^{B}$).

### 3.1. The Equilibria 

#### 3.1.1. Pure-Strategy Equilibria: 

The game has two combinations of strategies in which pure-strategy equilibria exist. The first is at Point A, where both players choose to cooperate fully; the second is at Point D, where they choose not to cooperate at all.

#### 3.1.2. Mixed-Strategy Equilibria:

a. For the government: mark the probabilities of the government’s adopting each of its strategies $q_{1}$, the probability of cooperation, and $q_{2}$, the complementary probability. Mark the utility to citizens of adopting each of their strategies $EU_{C}^{1},EU_{C}^{2}$:
  (5) $$EU_{C}^{1}=q_{1}*U_{C}^{A}+q_{2}*U_{C}^{C}$$
  (6) $$EU_{C}^{2}=q_{1}*U_{C}^{B}+q_{2}*0$$

Equate $EU_{C}^{1},EU_{C}^{2}$ and $q_{2}=1-q_{1}$.

(7) $$q_{1}*U_{C}^{A}+\left(1-q_{1}\right)*U_{C}^{C}=q_{1}*U_{C}^{B}$$

Hence:

(8) $$q_{1}=\frac{U_{C}^{C}}{U_{C}^{C}+U_{C}^{B}-U_{C}^{A}}$$

(9) $$q_{2}=1-\frac{U_{C}^{C}}{U_{C}^{C}+U_{C}^{B}-U_{C}^{A}}$$

b. For the citizens: mark the probabilities of citizens’ adopting each of their strategies $p_{1}$, the probability of being vaccinated, and $p_{2}$, the complementary probability. Mark the utility to the government of adopting each of its strategies $EU_{G}^{1},EU_{G}^{2}$.
  (10) $$EU_{G}^{1}=p_{1}*U_{G}^{A}+p_{2}*U_{G}^{B}$$
  (11) $$EU_{G}^{2}=p_{1}*U_{G}^{C}+p_{2}*0$$

Equate $EU_{G}^{1},EU_{G}^{2}$ and $p_{2}=1-p_{1}$:

(12) $$p_{1}*U_{G}^{A}+\left(1-p_{1}\right)*U_{G}^{B}=p_{1}*U_{G}^{C}$$

Hence:

(13) $$p_{1}=\frac{U_{M}^{B}}{U_{G}^{B}+U_{G}^{C}-U_{G}^{A}}$$

(14) $$p_{2}=1-\frac{U_{M}^{B}}{U_{G}^{B}+U_{G}^{C}-U_{G}^{A}}$$

### 3.2. Numerical Example

To demonstrate the model and its implications for the vaccination rate in an attempt to explain the differences that we found between the Bedouin populations of the north and the south, we construct a game matrix for each region (See Table 4 and Table 5) such that the numerical values of the utilities will reflect the differences in the underlying conditions of the game, namely, the differences in the long-term variables. For example, utilities $U_{G}^{C}$ and $U_{C}^{B}$ are larger in the game matrix of the northern region than in that of the southern region, for the reasons mentioned above.


(15) $$p_{1}=\frac{U_{M}^{B}}{U_{G}^{B}+U_{G}^{C}-U_{G}^{A}}=\frac{-2}{-2+5-6}=0.67$$

(16) $$q_{1}=\frac{U_{R}^{C}}{U_{C}^{C}+U_{C}^{B}-U_{C}^{A}}=\frac{-2}{-2+5-6}=0.67$$

As the example shows, there are two pure-strategy Nash equilibria at points A and D and there is also a mixed-strategy Nash equilibrium: *q* (0.67, 0.33), *p* (0.67, 0.33), and the utilities for the players are *u* (3.35, 3.35).


(17) $$p_{1}=\frac{U_{G}^{B}}{U_{G}^{B}+U_{G}^{C}-U_{G}^{A}}=\frac{-2}{-2+2-6}=0.33$$

(18) $$q_{1}=\frac{U_{R}^{C}}{U_{C}^{C}+U_{C}^{B}-U_{C}^{A}}=\frac{-2}{-2+2-6}=0.33$$

As the example shows, there are two pure-strategy Nash equilibria at points A and D as well as a mixed-strategy Nash equilibrium, *q* (0.33, 0.67) and *p* (0.33, 0.67); the utilities for the players are *u* (0.65, 0.65).

### 3.3. Results

The game matrices and equilibria obtained indicate that both the utility to citizens from being vaccinated (*p*_1_) and the probability of the government’s investing in cooperation (*q*_1_) are higher in the north than in the south. This is due to different underlying conditions in the respective areas; given the higher levels of trust and health literacy between citizens and government in the north, this outcome is consistent with the statistical data presented above.

Citizens are more trustful of the government in the northern region than in the southern; consequently, each side there trusts the other side to invest together in promoting health processes. The north also outperforms the south in health literacy. These two conditions find expression in the strategies that the players (*p*, *q*) choose and in the way the preferable strategy from each player’s standpoint is calculated. Indeed, the data gathered square with the outcomes of the model in which $p_{1}>p_{2},\;q_{1}>q_{2}$ in the north.

In the southern region, the combination of distrust between the sides and citizens’ poor health literacy yields a mirror image. This is manifested in $p_{1}<p_{2}$, which makes it the citizens’ preferred strategy to remain unvaccinated. The government, in turn, does not expect citizens to choose to vaccinate with high probability; therefore, it gives this strategy a lower probability, $q_{1}<q_{2}$.

## 4. Discussion

COVID-19 vaccination is crucial in controlling the pandemic and mitigating its impact on society [1,63]. It protects individuals from severe illness, reduces the spread of the virus, and mitigates hospitalization and death [2]. The findings of our study, based on Ministry of Health data and our in-depth interviews, present a clear picture of the low rate of COVID-19 vaccination among the Bedouin in southern Israel relative to those in the northern part of the country, and of the large disparity between both Bedouin collectivities and the country’s general population.

The first obstacle to vaccination and the prime determinant of the low vaccination rate among the southern part of the research population is scanty trust in the state and in some of its institutions—the government and its decision makers generally, including the Ministry of Health—and, more specifically, the information that this establishment gives the public [13].

This comportment is typical of conservative minority groups that suspect establishments and their good faith in policymaking toward their community. The southern Bedouin population maintains a complex relationship with the state and its authorities. An underlying land issue intensifies mutual distrust [13] and is accompanied by feelings of discrimination in resource allocation. Wherever the state and its citizens distrust each other, it is difficult to motivate citizens to act. This wariness projects saliently onto the low vaccination rate of the southern Bedouin.

The second obstacle that may explain the gap is poor health literacy and lack of access to information among the Bedouin in the south [45,46,47]. This finding corresponds with studies that investigated the relation between poor literacy and reluctance to receive COVID-19 vaccination among minority groups. Poor health literacy is also associated with the paltry education, scanty resources, economic hardships, and relatively conservative social structure of the southern Bedouin [64]. One of the determinants of willingness to be vaccinated against COVID-19 is concern about the safety of the vaccine, extraneous motives to encourage vaccination among this population, and an abundance of confusing information and guidelines, as evidenced in the interviews. The Israel State Comptroller, in a special report on how the Government of Israel coped with the pandemic [65], noted that the Ministry of Health began to provide information and messages in Arabic more gradually than it did information in Hebrew and that a disparity took shape between general Jewish society and Arab society in assimilating the behavioral guidelines.

The relationship between distrust in the government and poor health literacy is also found to have cultural aspects [33,64]. The disparities between north and south in vaccination rates underscore this point in a major way. The northern Bedouin have undergone processes of socialization and rapprochement with the values of modern Israeli society; this, by necessity, has influenced their COVID-19 vaccination rates. Still, the cultural gaps were evident in the interviewees’ remarks and the research literature. This was manifested in the prioritization of healthcare professionals’ and healthcare actions in accordance with tribal struggles and balances of power. Bedouin society’s health comportment is strongly influenced by the clan values on which it is based. Modernity and individualism are more common in the north; there, people make up their own minds [66]. 

Amid the other factors mentioned above, the informational disarray that prevailed abroad and in Israel, and all the more vis-à-vis special cultural groups, depressed vaccination rates among the southern Bedouin. The set of anti-COVID guidelines was deficient due to factors of surprise and inexperience in dealing with this virus [65]. Bedouin society in the south, some of which is unconnected to standard communication media, received the guidelines in a faulty manner or relied on information obtained from unqualified intra-community sources. Studies point to a strong connection between the mediator of information and the extent of trust in the information, contingent upon the popularity and credibility of the source [21].

In addition to these factors is the leadership crisis that all of Arab society in Israel is experiencing [33]. The Arab religious leadership lacks the authority it once had, making the population’s ability to trust religious leaders’ conveyance of messages insufficient as well. This defect definitely attenuated the establishment’s ability to create a channel of dialogue with this conservative minority community, necessitating the development of alternate channels in order to optimize action in the situation at hand.

By modeling on the basis of game-theory tools, we showed how these variables can change the equilibria in a game of non-cooperation versus cooperation between the government and citizens. The model offers a plausible explanation of the higher vaccination rates in the north and squares with the data gathered and the information harvested from the interviews.

Our use of a game-theory model yields meaningful outcomes that attest to the different long-term underlying states of the northern and southern Bedouin populations, placing these regions at different equilibria. In the northern region, trust between citizens and the state and its institutions provides solid grounds for an optimal outcome of cooperation between the sides. This outcome, of course, remains contingent upon government investment in the near term. Due to the auspicious underlying conditions in the north, the more this investment takes place, the more likely citizens are to accept vaccination.

In the south, in contrast, short-term investment of resources in problem solving will be less fruitful and may not be fruitful at all. The shaky underlying conditions of citizen-government relations in the south lead to an outcome of distrust in the game, in which no player has an incentive to invest effort for the public because it does not believe the other side will reciprocate. To improve cooperation and vaccination rates, one must solve long-term problems first. Investment is needed in building the trust of citizens in the south in the state and its institutions and improving interrelations between them, by means of fundamental longitudinal processes including many changes: investment in education, law enforcement, regularizing the status of unincorporated settlements, and so on. Absent these basic changes in bilateral relations, applying short-term solutions alone will not suffice.

## 5. Conclusion and Practical Implications

In this study, we sought ways of motivating conservative minority communities to cooperate with healthcare policy at times of crisis, with COVID-19 vaccination as the test case. The object of the study was the Bedouin society of Israel, differentiating between that in the south of the country and that in the north.

The summary of the findings from a broad perspective, based on the three main research tools that we chose, yields a clear picture that necessitates intervention and special planning by policymakers at the national level.

Since the variables of trust and level of health literacy figure prominently and meaningfully in the findings of the study, we recommend policymaking at two levels. First, solid trust needs to be established in the long term by recruiting members of the veteran community, who are strongly trusted and widely recognized by the population, for positions in the healthcare system. These figures would create a bridge of sorts between the citizens and the government and its authorities, helping to internalize the messages of the healthcare system in non-crisis situations and creating a triangle of strong trust. This strengthening of trust would be helpful in persuading citizens to internalize messages and cooperate in the course of crises—especially health crises, which evoke more meaningful apprehensions than would other matters.

At the second level, government policy on extreme health challenges and crises may be enhanced by understanding and recognizing the promotion and the more meaningful application of health literacy among conservative minority communities that have little such literacy. The advancement of health literacy among the Bedouin, mutatis mutandis for cultural and linguistic contents and the integration of professionals who belong to the community, would enhance the community’s access to information and build greater trust in the government and the healthcare system, thus minimizing the impact of “fake news”. 

## 6. Limitations of the Study and Future Directions of Research 

This study investigated obstacles to COVID-19 vaccination. Information about this specific vaccination remains deficient around the world and is accompanied by some suspicion among all population groups. Therefore, this study should be expanded to additional states of emergency and should investigate obstacles and attitudes toward additional situations. The set of communities investigated should be expanded to additional conservative minority collectivities of similar nature in other countries; a comparison among them would help to improve the accuracy of the model and its components.

## Figures and Tables

**Table 1:** Two-Sample t-test results, assuming equal variances.

	First Dose		Second Dose		Third Dose	
	South	North	South	North	South	North
Mean	0.26	0.63	0.22	0.56	0.08	0.29
Variance	0.02	0.00	0.02	0.00	0.00	0.00
Observations	10.00	10.00	10.00	10.00	10.00	10.00
Pooled Variance	0.01		0.01		0.00	
Hypothesized Mean Difference	0.00		0.00		0.00	
df	18.00		18.00		18.00	
t Stat	−7.32		−7.76		−7.98	
P (T ≤ t) one-tail	0.00		0.00		0.00	
t Critical one-tail	1.73		1.73		1.73	
P (T ≤ t) two-tail	0.00		0.00		0.00	
t Critical two-tail	2.10		2.10		2.10	

**Table 2:** Variables and parameters.

Parameter	Parameter Name	Definition
$U_{C}$	Citizens’ net utility	Net utility for the citizen
$V_{C}$	Citizens’ vaccination utility	The (subjective) utility for the citizens of the very act of vaccination
$U_{G}$	Government’s net utility	Net utility for the government
$V_{G}$	Government’s vaccination utility	The government’s utility from vaccinating citizens
$C_{C}$	Citizens’ vaccination costs	The cost to citizens of receiving vaccination
$C_{G}$	Government’s vaccination costs	The cost to the government of vaccinating the population
$T_{HP}$	Trust in healthcare professionals	Level of citizens’ trust in the Ministry of Health
$T_{G}$	Trust in government	Level of citizens’ trust in the government
$T=T_{HP}+T_{G}$	Total trust	Level of citizens’ trust in the government and in healthcare professional
$H_{L}$	Health literacy	Level of health literacy
$n_{v}$	Number of vaccinated citizens	The number of citizens vaccinated by the government

**Table 3:** The game matrix.

Government Citizens	Invests in the Process	Does Not Invest in the Process
Receive a COVID-19 vaccine	**A** $U_{C}^{A}$,$\;U_{G}^{A}$	C $U_{C\;}^{C}$,$\;U_{G}^{C}$
Do not receive a COVID-19 vaccine	B $U_{C\;}^{B}$,$\;U_{G}^{B}$	**D** 0,0

**Table 4:** Game matrices for the north.

Government Citizens	Invests in the Process	Does Not Invest in The Process
Receive a COVID-19 vaccine	**A** 6,6	C −2,5
Do not receive a COVID-19 vaccine	B 5,−2	**D** 0,0

**Table 5:** Game matrices for the south.

Government Citizens	Invests in the Process	Does Not Invest in the Process
Receive a COVID-19 vaccine	**A** 6,6	C −2,2
Do not receive a COVID-19 vaccine	B 2,−2	**D** 0,0

## Data Availability

Not applicable.
